# Autosis and autophagic cell death: the dark side of autophagy

**DOI:** 10.1038/cdd.2014.143

**Published:** 2014-09-26

**Authors:** Y Liu, B Levine

**Affiliations:** 1 Center for Autophagy Research, University of Texas Southwestern Medical Center, Dallas, TX 75390-9113, USA; 2 Department of Internal Medicine, University of Texas Southwestern Medical Center, Dallas, TX 75390-9113, USA; 3 Department of Microbiology, University of Texas Southwestern Medical Center, Dallas, TX 75390-9113, USA; 4 Howard Hughes Medical Institute, University of Texas Southwestern Medical Center, Dallas, TX 75390-9113, USA

## Abstract

It is controversial whether cells truly die via autophagy or whether — in dying cells — autophagy is merely an innocent bystander or a well-intentioned ‘Good Samaritan' trying to prevent inevitable cellular demise. However, there is increasing evidence that the genetic machinery of autophagy may be essential for cell death in certain settings. We recently identified a novel form of autophagy gene-dependent cell death, termed autosis, which is mediated by the Na^+^,K^+^-ATPase pump and has unique morphological features. High levels of cellular autophagy, as occurs with treatment with autophagy-inducing peptides, starvation, or *in vivo* during certain types of ischemia, can trigger autosis. These findings provide insights into the mechanisms and strategies for prevention of cell death during extreme stress conditions.

## Facts

- Although physiological levels of autophagy are essential for the maintenance of cellular homeostasis during various stress conditions, excessive or uncontrolled levels of autophagy are able to induce autophagy-dependent cell death.
- Autosis is an autophagy-dependent non-apoptotic form of cell death, characterized by enhanced cell substrate adhesion, focal ballooning of the perinuclear space, and dilation and fragmentation of endoplasmic reticulum.
- Autosis is triggered by autophagy-inducing peptides, starvation, and neonatal cerebral hypoxia-ischemia.
- Pharmacological inhibition or genetic inactivation of Na^+^,K^+^-ATPase blocks autosis *in vitro* and *in vivo*.

## Open Questions

- How is autosis initiated and executed?
- What are the biomarker(s) of autosis?
- Is autosis a subtype of autophagic cell death or does all autophagic cell death occur by autosis?
- Does autosis occur under other pathophysiological conditions?
- What are the biological and clinical implications of autosis?

Programmed cell death is a highly regulated cellular response in metazoans to control cell fate following various cellular stresses and/or extrinsic stimuli. Historically, three types of cell death have been identified based on morphological criteria, including Type I (apoptosis), Type II (autophagic cell death), and Type III (necrosis).^1,2^ Autophagic cell death was originally defined as a type of cell death accompanied by large-scale autophagic vacuolization of the cytoplasm and resultant vacuolated appearance. On the basis of these ultrastructural criteria, autophagic cell death was described during animal development, tissue homeostasis, and in diseased tissues, as well as in cultured mammalian cells treated with chemotherapeutic agents or other toxic compounds.^3^ Using more stringent genetic criteria to define a causative role of autophagy in cell death, several studies in the past decade have shown that autophagy-dependent cell death occurs under certain experimental conditions.^4^ Recently, we identified a novel form of autophagy gene-dependent, Na^+^,K^+^-ATPase-regulated, non-apoptotic cell death, termed ‘autosis', which is induced by autophagy-inducing peptides, starvation, and hypoxia-ischemia, and characterized by the disappearance of endoplasmic reticulum and focal swelling of the perinuclear space.^5^ In this review, we summarize our current understanding of autophagy-dependent cell death and autosis in mammalian systems.

## Historical Perspective of Autophagy and Autophagic Cell Death

**Figure 1:**
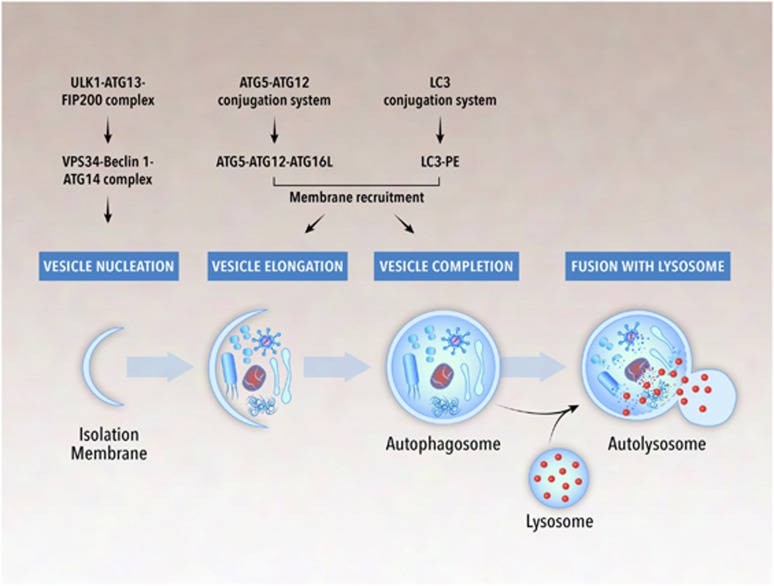
Schematic overview of the autophagy pathway. Autophagy is a catabolic process in which damaged or redundant cellular organelles or exogenous pathogens are degraded by autophagosomes. The formation of the autophagosome includes vesicle nucleation, vesicle elongation, and vesicle completion, which are tightly regulated by various autophagy-related proteins, some of which are listed in the figure. The mature autophagosome then fuses with the lysosome to form an autolysosome. Inside the autolysosome, the sequestered contents are degraded

Physiological levels of autophagy promote cellular survival in response to a variety of stress conditions, including starvation, hypoxia, mitochondrial damage, and pathogen infection.^6, 7, 8^ A set of evolutionarily conserved proteins, the autophagy-related (Atg) proteins, mediate the homeostasis function of autophagy through the formation of a double-membrane bound structure termed the autophagosome (Figure 1). In the initial stage (vesicle nucleation), the ULK/Atg1 complex activates the class III phosphatidylinositol 3 kinase complex, which recruits a series of Atg proteins to the isolation membrane (phagophore) that forms either from preexisting organelles^6^ or via *de novo* lipid synthesis.^9^ In the subsequent stages of vesicle elongation and completion, two ubiquitin-like conjugation systems (Atg12-conjugation and microtubule-associated protein 1A/1B light chain 3 (LC3)-conjugation system) govern the covalent conjugation of Atg5 to Atg12 and the conversion of LC3-I to its phosphatidylethanolamine-conjugated form (LC3-II).^10^ The outer membrane of the mature autophagosome then fuses with the lysosome to form an autolysosome, in which the sequestered cytoplasmic material is degraded.

Excessive levels of autophagy have been observed in association with various forms of cell death and the term ‘autophagic cell death' was originally generated to describe cell death associated with autophagy.^11^ However, the evidence linking autophagy to cell death in these early reports was largely circumstantial, as the morphological criteria for ‘autophagic cell death' indicated only that autophagy was present in dying cells but could not be used to determine whether autophagy had a causative role in the death process.

To avoid confusion, the Nomenclature Committee on Cell Death (NCCD) reintroduced the term ‘autophagic cell death' to describe cell death that is suppressed by inhibition of the autophagy pathway.^4^ As none of the presently available inhibitors of autophagy function exclusively in autophagy regulation, it became necessary to include genetic inactivation of essential autophagic proteins to establish the role of autophagy in cell death. Accordingly, the most recent guidelines from the NCCD specifies that autophagic cell death should be identified only if the process is blocked by genetic interventions targeting at least two components of the molecular machinery of autophagy.^4^ This requirement recognizes that many autophagy proteins have multiple autophagy-independent functions. Another requirement is that clonogenic survival assays should be used to demonstrate long-term protection against cell death with genetic inhibition of autophagy, to avoid misleading conclusions based solely on alterations of cell death kinetics.

Using these more stringent criteria to define a causative role of autophagy in cell death, several studies in the past decade have shown that autophagy can be mechanistically involved in cell death, including cell death in invertebrate development (e.g., *Drosophila* midgut degradation and salivary gland destruction) and hypersensitive cell death in plants.^12,13^ In cultured mammalian cells, autophagy can contribute to cell death under certain experimental conditions characterized by the absence of intact apoptosis pathways; these include etoposide- and staurosporine-induced death in *Bax*^−/−^; *Bak*^−/−^ murine embryonic fibroblasts,^14,15^ caspase inhibition in mouse fibroblasts,^16,17^ expression of a Beclin 1 mutant that escapes Bcl-2 regulation in breast cancer cells that have inactive caspase 3,^18^ and caspase 10 inhibition in myeloma cells.^19^ In cells competent for apoptosis, high levels of autophagy can also lead to autophagy gene-dependent and caspase-independent cell death, including in cells expressing a short isoform of p19^ARF^,^20^ in human ovarian epithelial cells expressing oncogenic H-Ras^V12^,^21^ and in cells exposed to a variety of environmental stresses and toxic agents.^22,23^

Although such studies provide genetic support for autophagy as a bona fide mode of cell death, the nature of autophagic cell death that occurs in mammalian cells and tissues in response to pathophysiological stimuli (such as starvation) has (until recently) remained poorly defined. Except for an accumulation of autophagosomes/autolysosomes, it has been unclear whether cells that die ‘by autophagy' have any unique morphological features or exclusive death machinery to distinguish autophagic cell death from apoptosis and necrosis. The only morphological feature that has been linked to autophagic cell death — autophagic vacuolization — is also commonly observed in cells undergoing apoptotic or necrotic cell death, and no proteins aside from the core autophagy proteins have been shown to be required for autophagic cell death.

## Links between Autophagy and other Death Machineries

Perhaps the best-characterized example of molecular overlap between regulation of autophagy and apoptosis is the interaction of the BH3 domain of the Beclin 1 autophagy protein with the anti-apoptotic proteins, Bcl-2 and Bcl-X_L_.^24^ In nutrition-rich conditions, the autophagy activity of Beclin 1 is inhibited by endoplasmic reticulum (ER)-localized Bcl-2.^18^ Cells possess multiple different mechanisms to positively and negatively regulate this interaction, and thereby either inhibit or stimulate autophagy. For example, the complex is stabilized by Mst1 kinase phosphorylation of the Thr108 residue in the BH3 domain of Beclin 1,^25^ and the complex dissociates after JNK1-mediated Bcl-2 phosphorylation (during nutrient starvation),^26^ DAPK-mediated phosphorylation of the Thr119 residue in the BH3 domain of Beclin 1,^27^ or overexpression of BH3-only proteins or treatment with BH3 peptidomimetic drugs.^28^ Moreover, disruption of the Bcl-2/Beclin 1 complex is required for starvation- and exercise-induced autophagy *in vivo*.^7,18^ The dual regulation of apoptosis and autophagy of Bcl-2 family members by binding to BH3 domains of pro-apoptotic proteins and pro-autophagy proteins most likely reflects the need for cells to coordinately regulate these two pathways; however, to date, there is no evidence that full-length Beclin 1 participates in apoptosis, and the practical implications of this dual regulation are not understood.

The apoptotic machinery may inhibit autophagy (presumably as a means of shutting off a pro-survival pathway at a time when it is clearly futile) by triggering caspase-mediated cleavage of Atg proteins, including Beclin 1,^29^ Atg4D,^30^ and Atg16L.^31^ In addition, the pro-apoptotic molecule, Bim, binds to and sequesters Beclin 1 on microtubules, which thereby inhibits autophagy.^32^ Conversely, the autophagy machinery may modulate apoptosis by autophagic degradation of apoptotic proteins.^33^ In TRAIL-resistant tumor cells, autophagy-mediated degradation of active caspase 8 prevents TRAIL-mediated apoptosis.^34^ In addition, some autophagy proteins may function directly as a pro-apoptotic factor to initiate apoptosis, such as a calpain cleavage product of Atg5^35^ and the unconjugated form of Atg12.^36^

Autophagy has recently been demonstrated to have a role in necroptosis. The combination of an mTOR and lysosomal inhibitor results in RIPK1- and oxidative stress-dependent necroptosis in human renal carcinoma cell lines,^37^ suggesting that autophagy may inhibit necroptosis via the degradation of RIPK1 and decreasing reactive oxygen species (ROS). Although autophagy also has a cytoprotective role in TCR stimulation-induced necroptosis in c-FLIP-deficient T cells,^38^ it contributes to rapamycin/obatoclax- and dexamethasone-induced necroptosis.^39^ Autophagy induction by obatoclax results in FADD/RIPK1/RIPK3 recruitment to the autophagosomal membranes by interaction with Atg5,^40^ suggesting that autophagy may promote necroptosis via the assembly of the necrosome on autophagosomes.

In addition to apoptosis and necroptosis, autophagy also regulates other death pathways, including immunogenic cell death, entosis, and pyroptosis. Suppression of autophagy results in diminished release of ATP from dying tumor cells, indicating an essential role of autophagy in immunogenic cell death.^41,42^ Downstream (e.g., Vps34, Atg5, and Atg7), but not upstream (e.g., ULK1, Atg13 and FIP200), autophagy components are involved in LC3 recruitment to single-membrane entotic vacuoles,^43^ suggesting that entosis may utilize autophagy proteins to facilitate the clearance of internalized cells by lysosomes. During *Shigella* infection, autophagy may have a protective role against pyroptosis as treatment with the autophagy inhibitor 3-MA enhances caspase-1-dependent cell death in infected macrophages.^44^

## Autosis, a New Form of Non-Apoptotic Cell Death

To investigate cell death in the setting of very high levels of autophagy, we evaluated the effects of an autophagy-inducing peptide, Tat-Beclin 1, on autophagy-dependent cell death. Tat-Beclin 1 is derived from 18 amino acids of the evolutionarily conserved domain of Beclin 1 fused to 11 amino acids from the HIV Tat protein transduction domain to facilitate cellular peptide entry. This peptide induces robust levels of autophagy through a mechanism thought to involve disruption of Beclin 1/GAPR-1 binding in the Golgi complex.^45^ Doses that have no apparent cytotoxic effects have a variety of beneficial effects *in vitro* and *in vivo* in mouse models; Tat-Beclin 1 inhibits the replication of HIV, arboviruses, and *Listeria monocytogenes in vitro*; enhances the clearance of mutant huntingtin protein aggregates; and protects mice against lethal chikungunya and West Nile virus infection.^45^ In fact, the brains of West Nile virus-infected mice treated with Tat-Beclin 1 have a marked reduction in neuronal cell death, as measured by TUNEL staining, presumably as a consequence of reduced central nervous system (CNS) viral titers and/or cytoprotective effects of the peptide.

Although these results suggest that autophagy upregulation may be beneficial and pro-survival in certain settings, we also found that — at least *in vitro* — Tat-Beclin 1-induced time- and dose-dependent cell death.^5^ This death was blocked by pharmacological or genetic inhibition of autophagy, but not of apoptosis or necroptosis; it did not overlap genetically with apoptosis or necroptosis; and it displayed unique morphological features. This autophagy-dependent type of cell death, termed ‘autosis' (*auto*, autophagic; *tosis*, death), is characterized by enhanced cell-substrate adherence, dilated and fragmented ER (early) and ER disappearance (late), and nuclear membrane convolution (early) and focal swelling of the perinuclear space (late; Table 1). We noted that another published autophagy-inducing peptide, Tat-vFLIP *α*2, which acts by releasing ATG3 from cellular FLIP,^46^ also induced autosis,^5^ indicating that autosis may be the underlying mechanism of cell death in previous reports of autophagic cell death.

**Figure 2:**
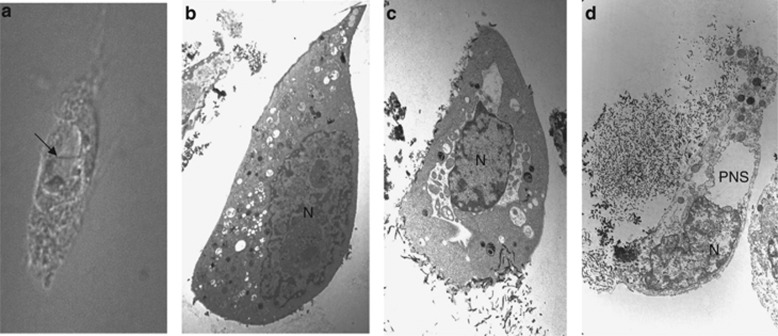
Morphological features of autosis. (**a**) Representative light microscopic image of an autotic HeLa cell during starvation. Arrow shows area of focal nuclear concavity with adjacent focal ballooning of perinuclear space. (**b**–**d**) Representative electron microscopic images of different stages of autotic cell death; including (**b**) a cell in an early stage of autosis (referred to as phase 1a) with nuclear membrane convolution, mild chromatin condensation, numerous autophagosomes, and autolyososomes, dilated and fragmented ER, and electron-dense mitochondria; (**c**) a cell in a mid-stage of autosis (referred to as phase 1b) with separation of the inner and outer nuclear membrane, the presence of membrane-bound densities in the perinuclear space; and (**d**) a cell in the final stage of autosis (referred to as phase 2) with focal nuclear concavity, focal ballooning of the perinuclear space (which is empty) and disappearance of cellular organelles such as ER, autophagosomes, and autolysosomes. N, nucleus; PNS, perinuclear space

Autotic death can be identified by several criteria, including: (1) the absence of morphological, biochemical, and genetic evidence for other cell death pathways, (2) unique morphological changes, and (3) a unique dependence on Na^+^,K^+^-ATPase. While autosis is accompanied by several stereotypic morphological features (some of which have already been described in ‘classical' type II autophagic cell death; Table 1), the ‘sine qua non' of autosis is the nuclear membrane changes (Figure 2). At the light microscopic level, there is nuclear shrinkage with a focal concave portion adjacent to a round, vacuole-like entity (with a ‘balloon' appearance; Figure 2a); at the ultrastructural level, this balloon appearance corresponds to focal separation of the inner and outer nuclear membranes (Figures 2c and d). Although both autosis and apoptosis demonstrate chromatin condensation, it is very mild in autosis (Figure 2b) compared with apoptosis and neither DNA laddering nor TUNEL-positive staining occurs in autosis.^5^ Another unique feature of autosis compared with other forms of cell death is the increased substrate adherence of dying cells.

In addition to the unique nuclear membrane changes and increased substrate adherence, autosis demonstrates a distinct biochemical mechanism of regulation. Autotic cell death, but not apoptosis or necrosis, is inhibited by pharmacological or genetic inhibition of Na^+^,K^+^-ATPase.^5^ Conversely, treatment with caspase or RIP kinase inhibitors or genetic deletion of pro-apoptotic Bax and Bak or pro-necroptotic RIPK1 and RIPK3 does not protect cells against autotic cell death.^5^ Unlike necrosis or necroptosis, ROS is not a mediator in autosis.^5^ Thus, autosis utilizes an exclusive death machinery to initiate or execute cell death that is distinct from that of previously described forms of cell death. However, no biochemical markers are currently available to identify cells dying by autosis.

Although autosis was initially identified in the context of high levels of autophagy triggered by an autophagy-inducing peptide, this death pathway also occurs naturally during certain stress conditions. *In vitro*, autosis occurs in a subpopulation of cells that undergo the highest levels of autophagy and become substrate-adherent during nutrient starvation.^5^ Moreover, *in vivo*, autotic cell death occurs in hippocampal CA3 region neurons of neonatal rats subjected to cerebral hypoxia-ischemia.^5^ The unique features of autosis may have been missed in other tissues with ischemia-induced autophagic cell death that lack features of apoptosis and necrosis. More broadly, it remains to be determined whether autosis represents a subtype of autophagic cell death or whether all bona fide cell death by autophagy occurs through an autotic route.

## How Do Cells Die in Autosis and Autophagic Cell Death?

In a chemical screen of ~5000 compounds with known biological targets, cardiac glycosides, antagonists of Na^+^,K^+^-ATPase, were the only identified inhibitors of autosis.^5^ Cardiac glycosides rescue clonogenic survival *in vitro* of cells that die by autophagy-inducing peptide or starvation-induced autosis, and they also blocks hippocampal autotic death and decrease cerebral infarct size following neonatal rat carotid artery ligation. In addition, knockdown of the *α*-subunit of Na^+^,K^+^-ATPase protects cells against autosis,^5^ confirming the specificity of the target of cardiac glycosides. The magnitude of autotic death rescue with cardiac glycosides or Na^+^,K^+^-ATPase knockdown is greater than with single autophagy gene knockdown.^5^ Although this may reflect incomplete ablation of the autophagy pathway by autophagy gene knockdown, it is also possible that autophagy proteins do not function in the core death machinery of autosis. Rather, excessive levels of autophagy may act upstream as a primer to ignite the death machinery that occurs in autosis.

**Figure 3:**
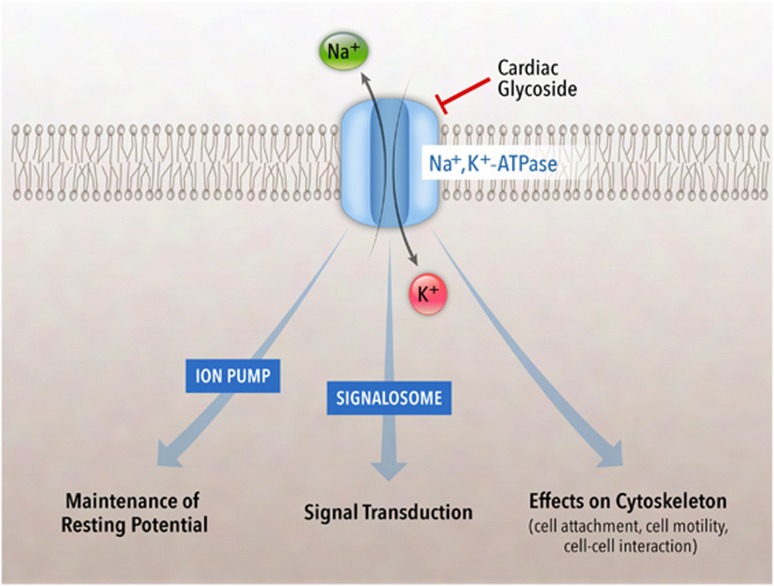
Multiple functions of Na^+^,K^+^-ATPase. Na^+^,K^+^-ATPase is a ubiquitous plasma membrane protein complex (consisting of *α-* and *β-*subunits) which functions in ion homeostasis by pumping Na^+^ out of cells and pumping K^+^ into cells. The ion pump function of Na^+^,K^+^-ATPase maintains the cell membrane potential, which is specifically inhibited by cardiac glycosides. In addition to its ion transporter function, Na^+^,K^+^-ATPase functions as a signalosome, recruiting diverse signaling molecules to initiate a series of signaling pathways. Na^+^,K^+^-ATPase also regulates the cytoskeleton, tight junctions, cell motility, and cell polarity

Na^+^,K^+^-ATPase is a well-characterized membrane pump that generates Na^+^ and K^+^ gradients across plasma membranes^47^ and also functions as a versatile transducer to mediate various cellular signaling pathways^48^ (Figure 3). In addition, Na^+^,K^+^-ATPase has a role in cell-substrate adhesion,^49,50^ which may explain the enhanced substrate adherence of cells undergoing autotic death. Na^+^,K^+^-ATPase localizes to the inner nuclear membrane^51^ as well as to the plasma membrane; nuclear envelope-associated Na^+^,K^+^-ATPase activity may alter membrane ionic transport and osmolarity, and thereby, contribute to the ER and perinuclear space expansion observed in autotic cells.

The distinct cellular morphological features of autosis may provide clues regarding the mechanisms involved in autotic death. The most striking morphological feature of autosis, separation of inner and outer nuclear membrane and resultant focal expansion of the perinuclear space, is rescued by cardiac glycosides or Na^+^,K^+^-ATPase knockdown. Before the final stage of cellular demise (when this perinuclear space is empty), there are several ~100-nm-sized membrane-bound densities in between the inner and outer nuclear membrane (Figure 2c); the origin of these structures is not known, but their presence is suggestive of dynamic membrane mobilization of the ER/outer nuclear membrane during autosis. Although similar expansion of the perinuclear space and dilation of the ER has not been previously reported in mammalian cell death, it has been observed in cells expressing disease-associated inner nuclear membrane lamin B receptor mutants.^52^ The molecular mechanism of this cellular phenotype was not defined, but it is noteworthy that expression of mutant sterol reductases localized to the ER and outer nuclear membrane, TM7SF2 and DHCR1, result in a similar phenotype as that of disease-associated lamin B mutants.^52^ This suggests that disruption of cholesterol metabolism in the ER/outer nuclear membrane is sufficient to produce the phenotype of ER and focal perinuclear space expansion. This phenotype may be caused by alterations of ER membrane properties including transport or channel conductance, which would result in osmotic changes and disruption of signaling through the nuclear envelope. In fact, members of the LINC complex, which links the outer and inner nuclear membranes to establish nucleocytoplasmic communication, are lost from the perinuclear expansions in cells expressing abnormal levels of sterol reductase.^52^ Interestingly, in plant wounds, there is an accumulation of phytoecdysteroids (e.g., 20-hydroxyecdysone),^53^ which is associated with cell death that has rapid nuclear collapse and nuclear envelope separation.^54^ In the *Drosophila* salivary gland, the steroid hormone 20-hydroxyecdysone induces autophagy-dependent cell death.^55^ Together, these observations suggest that disturbances in cholesterol metabolism and ER membrane homeostasis may contribute to autosis.

Given the crucial role of the ER in autophagosomal biogenesis,^56^ we speculate that stimulation of very high levels of autophagy in certain contexts may perturb the normal homeostatic mechanisms of the ER, leading to similar expansions of the ER lumen and perinuclear space as that seen with overexpression of mutant sterol reductases. Alternatively, the outer nuclear membrane may serve as a key site of initiation of the autosis program. The outer nuclear membrane is physically contiguous with the ER, but under physiological conditions, the autophagy machinery selectively avoids using the outer nuclear membrane as a membrane source to form autophagosomes. In the setting of extremely high levels of autophagy, however, the ER membrane may become exhausted as a source of autophagosomes, triggering the pathological use of the outer nuclear membrane (and perhaps some as-of-yet undefined signal that the cell should self-destruct). This hypothesis is consistent with the depletion of the ER at late stages of autosis and the presence of membrane-bound structures in the perinuclear space.

Although ROS may contribute to other forms of autophagy-dependent cell death,^13,16,17,57^ they do not appear to contribute to autosis (our unpublished data). Furthermore, other reported factors in autophagic cell death, including JNK,^14,15,58^ AMPK,^59^ MAPK,^60^ BNIP3,^61^ Noxa,^21^ cathepsin L,^62^ and BCLAF1^19^ have been demonstrated to participate in apoptosis or other cellular stress responses, suggesting that the crosstalk between apoptosis and autophagy has important regulatory functions in autophagic cell death. However, it is not clear whether these mechanisms induce or mediate autosis. Moreover, it is not known which cellular alterations function directly as inducers and executors of autosis and other forms of autophagic cell death; the possibilities include alterations in lipid or membrane dynamics, protein functions, ion homeostasis, ROS or other factors.

A Na^+^,K^+^-ATPase-mediated function is an essential upstream component of autosis; however, it is not known how this ion pump leads to the downstream morphological changes and demise of the cell. Na^+^,K^+^-ATPase may also function upstream of other death pathways, as neonatal rat treatment with cardiac glycosides blocks not only autotic death in the CA3 region of the hippocampus, but also other forms of cell death that occur in other regions of the brain.^5^ Despite recent evidence that autophagy, in the absence of other death pathways, can lead to cell death, it remains unknown whether the ultimate demise of the cell is a function of uncontrolled autophagy (i.e., eating oneself to death) or a byproduct of other cellular processes that occur as a consequence of autophagy. In autosis, the morphological features, including the late disappearance of the ER, are consistent with a scenario in which cells deplete organelles essential for cell survival through excessive autophagy. However, the dependence of this death process on the Na^+^,K^+^-ATPase pump favors a model in which autophagy and Na^+^,K^+^-ATPase activity cooperate to trigger an as-of-yet undefined death execution pathway.

Indeed, the concept that distinct signals from those involved in autophagy may be crucial in determining whether autophagy results in cell death has been previously demonstrated in *Dictyostelium discoideum*.^63^ The differentiation factor, DIF-1, triggers the death of starved cells undergoing autophagy, but not of non-starved cells, and conversely, starvation and autophagy, in the absence of DIF-1, do not result in cell death. An intriguing new frontier in autophagy and cell death research will be to more precisely define the spectrum of ‘second signals' for autophagic cell death, and how such signals interface with the autophagic machinery to result in cellular demise.

## Cardiac Glycosides and Na^+^K^+^-ATPase

Cardiac glycosides, a large family of naturally derived steroidal compounds, were first described for the treatment of heart failure in 1785,^64^ and ~50 years ago, the cellular target of cardiac glycosides was identified as Na^+^,K^+^-ATPase (Figure 3). Cardiac glycosides bind to the extracellular site of the *α*-subunit of Na^+^,K^+^-ATPase, which results in a conformational change of Na^+^,K^+^-ATPase and stabilizes the enzyme in a specific enzymatic conformation (E2-P).^65^ Inhibition of the pumping function of Na^+^,K^+^-ATPase by cardiac glycosides increases intracellular sodium concentrations, which inactivates Na^+^/Ca^2+^ exchange and causes an increase of intracellular calcium concentrations, and thereby a positive inotropic effect in cardiac muscle. Independently of this action, cardiac glycosides directly activate the Na^+^,K^+^-ATPase-mediated signalosome complex,^48,64,66^ which is subsequently transduced by the MAPK cascade and IP_3_ receptor to generate ROS, Ca^2+^ oscillations, and other cellular stresses. Na^+^,K^+^-ATPase also modulates cell polarity, cell motility, the cytoskeleton, and cell–cell interactions.^67,68^ It is unclear which of these actions of Na^+^,K^+^-ATPase are important in autosis.

The Na^+^,K^+^-ATPase is a heterodimeric protein consisting of a large *α* and a heavily glycosylated *β*-subunit.^69^ The *α*-subunit has four isoforms; *α*1 is ubiquitously expressed, whereas *α*2, *α*3, and *α*4, exhibit tissue-specific expression. However, it should be noted that, although all human *α*-isoforms are sensitive to cardiac glycosides, the *α*1 isoform in rodents is resistant to cardiac glycosides.^70^ Consistent with this species difference, autosis in mouse fibroblasts (which express predominantly the *α*1 isoform) is inhibited by siRNA knockdown of the Na^+^,K^+^-ATPase *α*1 subunit but not by treatment with the cardiac glycosides.^5^ However, as rodent brain expresses cardiac glycoside-sensitive *α*2 and *α*3 isoforms, treatment with the CNS-penetrating cardiac glycoside, neriifolin, prevents autotic neuronal cell death in rat cerebral hypoxic-ischemic injury.^5^

Cardiac glycosides modulate autophagy in different experimental settings. In a high-content screen for autophagy inducers, cardiac glycosides were found to induce autophagy under nutrition-rich conditions.^71^ In addition, cardiac glycosides induce autophagy in human non-small cell lung cancer cells through mTOR and ERK1/2 signaling pathways.^72^ However, in these studies, there was no genetic evidence that the autophagy-inducing effect of cardiac glycosides were due to Na^+^,K^+^-ATPase inhibition. In contrast, cardiac glycosides reduce levels of autophagy under autosis-inducing conditions, including treatment with the autophagy-inducing peptides, starvation, and cerebral hypoxia-ischemia.^5^ These data are consistent with dual roles of cardiac glycosides to maintain levels of autophagy within a physiological range.

Of note, animals increase circulating levels of endogenous inhibitors of Na^+^,K^+^-ATPase inhibitors (for example, ouabain-like factors) following various physiological and pathological stresses. It is intriguing to speculate that these endogenous Na^+^,K^+^-ATPase inhibitors allow animals to maintain proper physiological levels of autophagy by restraining excessive levels that may maladaptively promote autosis.

## Clinical Implications

Although the underlying mechanisms of the Na^+^,K^+^-ATPase-mediated autosis remain to be determined, the discovery of this unique form of cell death may have important clinical implications. Ischemic stroke, the destruction of cerebral tissue following deprivation of oxygen- and nutrient-rich blood flow, is an important cause of neurological deficits.^73^ Because the CNS cannot regenerate, the protection of neurons against ischemia-induced cell death is a key target in stroke therapy.

Multiple forms of cell death have been observed in neonatal ischemic brain damage,^74^ but neurons in CA3 regions of the hippocampus have a preference for an exclusive form of ischemia-induced cell death, which is characterized by early autophagic features in the absence of any signs of apoptosis or necrosis.^75^ Inhibition of autophagy by a pharmacological inhibitor, 3-methyladenine,^76,77^ or genetic inactivation of the autophagy genes, *Atg7* or *beclin 1*,^78, 79, 80^ significantly protects against ischemia-induced neuronal death, indicating that autophagy contributes to cell death and infarct size in neonatal ischemic brain injury. Furthermore, the cardiac glycoside, neriifolin, protects against neuronal injury in a mouse brain slide-based model^81^ and whole-animal studies.^5,81, 82, 83, 84^ These observations suggest that CNS-penetrating cardiac glycosides may warrant investigation in neonatal cerebral hypoxia-ischemia.

Autosis may contribute to ischemic injury in other organs, such as the heart and kidney. The induction of autophagy and cardiac injury during ischemia reperfusion is significantly attenuated in *beclin 1*^+/−^ mice,^85^ suggesting that autophagy may function as a pro-death factor in this setting. In mouse renal ischemia/reperfusion injury, *Atg5* deletion sensitizes the kidneys to ischemic injury,^86, 87, 88^ but autophagy has been shown to be detrimental in prolonged ischemia.^89, 90, 91, 92, 93, 94^ However, it is not clear that reperfusion injury is an important mechanism of cardiac dysfunction in the clinical setting, and there is no clinical evidence that cardiac glycosides are beneficial in preventing the consequences of cardiac or renal ischemia.

Although autophagy has a crucial role in degrading aggregate-prone proteins, in a mouse model of familial amyotrophic lateral sclerosis (ALS), autophagy induction by rapamycin increases motor neuron degeneration^95^ and *beclin 1* haploinsufficiency prolongs life span of mutant SOD1 transgenic mice,^96^ suggesting that autophagy may contribute to neurodegeneration in certain settings. If autotic cell death mediates neuronal cell death in mouse models of ALS, clinical trials of cardiac glycosides in patients with ALS may be warranted.

## Summary

**Figure 4:**
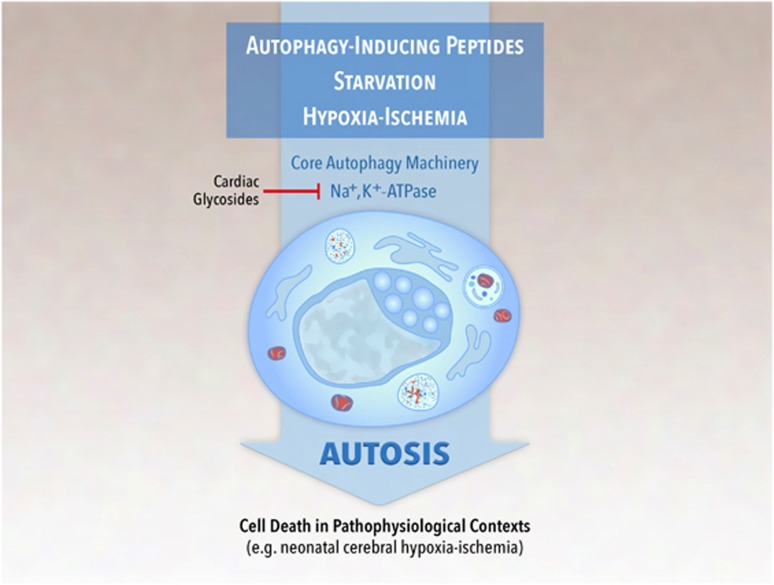
Summary of autosis inducers, mediators, and known pathophysiological contexts. Autosis is a novel form of autophagy-dependent, non-apoptotic cell death, which is induced by autophagy-inducing peptides, starvation, and hypoxia-ischemia. This process requires the core autophagy machinery and Na^+^,K^+^-ATPase. Thus far, it is known to contribute to cerebral infarct size in rodent neonatal cerebral hypoxia-ischemia. Cardiac glycosides, antagonists of Na^+^,K^+^-ATPase, inhibit autosis induced by peptides, starvation, and hypoxia-ischemia, and reduce cerebral infarct size in rodent neonatal cerebral hypoxia-ischemia

We identified a novel form of non-apoptotic autophagy-dependent cell death, termed autosis, which has unique morphological features; depends on the cellular Na^+^,K^+^-ATPase; and occurs during starvation, autophagy-inducing peptide treatment, and *in vivo* during cerebral hypoxia-ischemia (Figure 4). The discovery of autosis should stimulate exploration of the molecular mechanisms of this form of cell death; will pave the road to understanding the role of autophagic cell death under various physiological and pathophysiological conditions; may define a role for autosis in human disease; and is likely to provide novel targets for therapeutic strategies. Thus, vis-à-vis our understanding of autosis, it seems that death is only the beginning.

## Figures and Tables

**Table 1:** Comparison of the morphological features of autosis, ‘classical' type II autophagic cell death, apoptosis, and necrosis

**Cell death**	**Autosis**	**‘Classical' Type II Autophagic cell death**	**Apoptosis**	**Necrosis**
Plasma membrane	Focal plasma membrane rupture	Plasma membrane rupture; Blebbing sometimes observed	Blebbing of the plasma membrane with preserved integrity	Plasma membrane rupture
Nucleus	Nuclear membrane convolution and shrinkage; focal concavity of the nuclear surface; focal ballooning of perinuclear space	Minor changes	Nuclear compaction and fragmentation	Dilatation of the nuclear membrane
Chromatin	Mild-moderate chromatin condensation	Minor changes	Marked chromatin condensation	Mild-moderate chromatin condensation
Autophagic structures	Numerous autophagosomes and autolysosomes (early-mid stages) with disappearance in final stages	Numerous autophagosomes and autolysosomes	Varies	Varies
Other cytoplasmic organelles	Electron-dense mitochondria with abnormal internal structure (early); swollen mitochondria (late); dilated and fragmented ER (early) and ER disappearance (late)	Enlargement of Golgi, mitochondria, and ER sometimes observed; depletion of cytoplasmic organelles sometimes observed	Minor changes	Swelling
Other unique features	Enhanced cell-substrate adhesion; membrane-bound densities in perinuclear space		Rounding up of cells and detachment from substrate; formation of apoptotic bodies	Cell swelling

## Abbreviations

Atg: autophagy related
LC3: microtubule-associated protein 1A/1B light chain 3
NCCD: Nomenclature Committee on Cell Death
ER: endoplasmic reticulum
PNS: perinuclear space
ROS: reactive oxygen species
CNS: central nervous system
ALS: amyotrophic lateral sclerosis
